# Supplementary material for: Identification of stem rust resistance genes in wheat cultivars in China using molecular markers
Source: PeerJ. 2018 May 24;6:e4882. doi: 10.7717/peerj.4882 (PMC5971096; doi:10.7717/peerj.4882)
Supplement: Supplemental Information 1 [file peerj-06-4882-s001.zip › RAW data.doc]

Fig. 3A

Fig. 3B

Fig. 3C

Fig. 3D

Fig. 3E

Fig. 3F
